# Potential of PEGylated Toll-Like Receptor 7 Ligands for Controlling Inflammation and Functional Changes in Mouse Models of Asthma and Silicosis

**DOI:** 10.3389/fimmu.2016.00095

**Published:** 2016-03-11

**Authors:** Tatiana Paula Teixeira Ferreira, Lívia Lacerda Mariano, Roberta Ghilosso-Bortolini, Ana Carolina Santos de Arantes, Andrey Junior Fernandes, Michelle Berni, Valentina Cecchinato, Mariagrazia Uguccioni, Roberto Maj, Alcide Barberis, Patricia Machado Rodrigues e Silva, Marco Aurélio Martins

**Affiliations:** ^1^Laboratory of Inflammation, Oswaldo Cruz Institute, FIOCRUZ, Rio de Janeiro, Brazil; ^2^Institute for Research in Biomedicine, Universitá della Svizzera Italiana, Bellinzona, Switzerland; ^3^Telormedix SA, Bioggio, Switzerland

**Keywords:** TLR7, PEGylated ligands, asthma, ALI, silicosis

## Abstract

Prior investigations show that signaling activation through pattern recognition receptors can directly impact a number of inflammatory lung diseases. While toll-like receptor (TLR) 7 agonists have raised interest for their ability to inhibit allergen-induced pathological changes in experimental asthma conditions, the putative benefit of this treatment is limited by adverse effects. Our aim was to evaluate the therapeutic potential of two PEGylated purine-like compounds, TMX-302 and TMX-306, characterized by TLR7 partial agonistic activity; therefore, the compounds are expected to induce lower local and systemic adverse reactions. *In vitro* approaches and translation to murine models of obstructive and restrictive lung diseases were explored. *In vitro* studies with human PBMCs showed that both TMX-302 and TMX-306 marginally affects cytokine production as compared with equivalent concentrations of the TLR7 full agonist, TMX-202. The PEGylated compounds did not induce monocyte-derived DC maturation or B cell proliferation, differently from what observed after stimulation with TMX-202. Impact of PEGylated ligands on lung function and inflammatory changes was studied in animal models of acute lung injury, asthma, and silicosis following Lipopolysaccharide (LPS), allergen (ovalbumin), and silica inhalation, respectively. Subcutaneous injection of TMX-302 prevented LPS- and allergen-induced airway hyper-reactivity (AHR), leukocyte infiltration, and production of pro-inflammatory cytokines in the lung. However, intranasal instillation of TMX-302 led to neutrophil infiltration and failed to prevent allergen-induced AHR, despite inhibiting leukocyte counts in the BAL. Aerosolized TMX-306 given prophylactically, but not therapeutically, inhibited pivotal asthma features. Interventional treatment with intranasal instillation of TMX-306 significantly reduced the pulmonary fibrogranulomatous response and the number of silica particles in lung interstitial space in silicotic mice. These findings highlight the potential of TMX-306, emphasizing its value in drug development for lung diseases, and particularly silicosis.

## Introduction

Inhalation of environmental airborne substances in the form of aeroallergens and particulate matter can result in allergic respiratory dysfunctions and pneumoconiosis, such as asthma and silicosis, respectively (1–4). Moreover, air pollutants may impact on allergic airway-related morbidity and mortality (5, 6). While asthma is among those diseases with an obstructive pulmonary function pattern (7), silicosis is pathologically characterized as a fibrogranulomatous disease with a restrictive pulmonary function profile (8). Both asthma and silicosis are highly prevalent worldwide, cause elevated socioeconomic costs, and can be fatal (3). Steroidal anti-inflammatory agent combined to bronchodilators is the best way of controlling asthma currently, but glucocorticoid resistance and adverse effects limit the efficacy of this treatment (2). The situation is even more alarming in case of silicosis, since no proper therapy is available (9).

Pulmonary inflammation is central in these diseases. In asthma, inflammation is driven by the adaptive arm of host immunity and reflects an aberrant immune response specifically against otherwise harmless environmental factors in genetically predisposed individuals (2). Yet, the basis of the inflammatory response mounted following exposure to occupational air ­pollutants, such as crystalline silica particles, remains poorly understood (9). What is well established for both diseases, however, is the crucial role displayed by the airway wall as an immune-privileged innate barrier in which interdigitated dendritic cells (DCs), with the help of macrophages and epithelial cells, sense and respond to antigens, pollutant particles, and infectious microorganisms that traffic into the lung (10, 11). Upon intrusion, pathogens are recognized by pattern recognition receptors, and among them scavenger receptors (12–14) and Toll-like receptors (TLRs) play a pivotal role (15–18).

Toll-like receptors are located on the plasma membrane (TLR1, 2, 4, 5, 6, and 10) and endosomal/lisosomal vesicles (TLR3, 7, 8, 9, 11, 12, and 13) of immune cells (17). In humans, TLR1–10 are expressed by DCs, monocytes, macrophages, T cells, and B cells, and play important roles in their task of sensing and responding to “danger signals” presented by pathogens (19). All TLRs signal through the myeloid differentiation factor 88 (MyD88) adapter, with the exception of TLR3 that depends on TIR domain-containing adaptor inducing IFNβ (TRIF) (20–22). No detectable signaling occurs through TLRs in the absence of MyD88 and TRIF (20, 21). Within the airways, activation of TLR7 decreases adaptive response in a mechanism associated with upregulation of type 1 interferon (15, 16, 23). Moreover, TLR7 rapidly relaxes human airways (24).

Several synthetic small TLR7 agonists have been studied for their potential use to treat asthma (25, 26), but with limited benefits because of local and systemic inflammatory reactions (27–29). More recent investigations indicate that the conjugation of TLR7 ligands with a 6-unit oligo-ethylene glycol (PEG) moiety showed potential to inhibit the course of inflammatory diseases, such as diabetes, with retained TLR7 specificity and attenuated non-specific inflammation (30, 31). We hypothesize that PEGylated TLR7 partial agonists have potential to control not only allergic inflammatory lung diseases but also pneumoconiosis, with minimized adverse side effects. Hence, this study was undertaken in order to assess the impact of treatment with two PEGylated purine-like compounds, TMX-302 and TMX-306, upon pulmonary inflammation and function changes triggered by Lipopolysaccharide (LPS), allergen, or silica particles in mice. TMX-302 was previously planned by linking a specific TLR7 ligand, 9-benzyl-8-hydroxy-2-(2-methoxyethoxy) adenine (1V136), to a six-unit polyethylene glycol (PEG) (30), whereas, TMX-306 resulted from a molecular simplification of TMX-302.

## Materials and Methods

### Reagents

The PEGylated TLR7 ligands TMX-302 [3-(1-(1-(4-((6-amino-8-hydroxy-2-(2-methoxyethoxy)-9H-purin-9-yl)methyl)phenyl)-1-oxo-5,8,11,14,17,20-hexaoxa-2-azadocosan-22-yl)-1H-1,2,3-triazol-4-yl) propanoic acid] (MW = 791) and TMX-306 [1-(4-((6-amino-2-(2-methoxyethoxy)-8-oxo-7H-­purin-9(8H)-yl) methyl) phenyl)-1-oxo-5,8,11,14,17,20-hexaoxa-2-azatricosan-23-oic acid] (MW = 695), as well as the reference compound TMX-202 [2-(4-((6-Amino-2-(2-methoxyethoxy)-8-oxo-7H-purin-9(8H)-yl) methyl) benzamido) ethyl 2,3-Bis (dodecanoyloxy) propyl phosphate] (MW = 920) were provided by Telormedix (Bioggio, CH). LPS (strain *Escherichia coli* O127:B8), ovalbumin (OVA) (grade V), and crystalline silica particles were purchased from Sigma Chemical, St. Louis, MO, USA. All the others were obtained as further indicated.

### Animals

Male A/J and Swiss Webster mice (18–20 g) were obtained from the Oswaldo Cruz Foundation breeding colony and housed in standard laboratory cages at 22–25°C, on a 12 h light/dark cycle, and fed with food and water *ad libitum*. All the protocols involving animal care and use were approved by the Animal Ethics Committee of the Oswaldo Cruz Institute (License L-030/2015). C57BL/6 mice, purchased from Harlan (Italy), were maintained in the animal facility of the Institute for Research in Biomedicine, and all procedures were approved by the veterinarian authorities from the local committee (Comitato Etico Cantonale del Ticino, Switzerland) with the authorization number TI17/2010.

### Human Cell Isolation and Stimulation

Human PBMCs were isolated from buffy-coats (Central Laboratory of the Swiss Red Cross, Basel, Switzerland) using Ficoll-hypaque density centrifugation. Monocytes were isolated from PBMCs using CD14-magnetic beads (Miltenyi Biotec) and monocyte-derived dendritic cells (mo-DCs) generated *in vitro*, as previously described (32). Briefly, CD14^+^ cells were cultured for 4 days in complete medium supplemented with GM-CSF and IL-4 to induce mo-DCs differentiation. At day 4, medium was completely washed out, and cells were treated for 24 h with 10 μM of the indicated PEGylated TLR7 agonists or with vehicle only. At day 5, mo-DCs supernatant was collected to quantify the production of pro-inflammatory cytokines by stimulated cells, and mo-DCs were stained for maturation markers. Total PBMCs were cultured for 24 h in complete medium, supplemented with different concentrations of PEGylated TLR7 agonists (1 or 10 μM), and the supernatant was collected to quantify pro-inflammatory cytokine release.

### Cytokines Detection in Cell Supernatant

The concentration of IL-1β, IL-6, IL-8, IL-10, IL-12, and TNF in the supernatant of human PBMCs and human mo-DCs was determined using the BD™ cytometric bead array (CBA) (human inflammatory cytokine kit – 551811, BD Biosciences), according to manufacturer’s instructions.

### Flow Cytometric Analysis

For surface staining of human specimens, cell suspensions were incubated with the appropriate combination of the following monoclonal antibodies: CD19-PC5 (J3-119, Beckman Coulter), CD80-Brilliant Violet 421™ (2D10, BioLegend^®^), CD83-APC (HB15e, BioLegend^^®^^), CD86-APC (IT2.2, BioLegend^®^), and HLA-DR-V500 (G46-6, BD Horizon™). For surface staining of mouse specimens, cell suspensions were incubated with Fc-blocking antibody (Bioxcell, 2.4G2) to avoid unspecific Fc-Receptor binding. After washing, the cells were incubated with the appropriate combination of the following ­antibodies: CD11b-PECy7 (M1/70, BioLegend^®^), Ly6G-PE (1A8, BD Biosciences), Ly6C-Biotin (AL-21, BD Biosciences), CD3-APC(17A2, BioLegend^®^), CD45R-B220-PerCP-Cy5.5 (RA3-6B2, eBiosciences). To detect anti-Ly6C-Biotin antibody binding, cells were subsequently stained with streptavidin-FITC (Dako). The samples were acquired with BD LSRFortessa (BD Biosciences), and results were analyzed with FlowJo software (Tree Star, Inc.).

### B Lymphocytes Proliferation

Total human PBMCs were stained with 5 μM carboxyfluorescein succinimidyl ester (CFSE), using CellTrace™ CFSE Cell Proliferation kit (Invitrogen Molecular Probes, C34554), according to manufacturer’s instructions, and cultured in complete medium supplemented with 10 μM of the indicated PEGylated TLR7 agonists for 4 days. At day 4, B lymphocytes were stained with an anti-CD19 antibody, and proliferation was assessed by CFSE dilution.

### Leukocyte Mobilization from Bone Marrow

Age- and sex-matched mice (6–8 weeks) were randomly assigned to two groups, which were injected intraperitoneally with sterile saline (*n* = 5) or 200 nmoles of TMX-306 (*n* = 5). After 24 h, mice were sacrificed, and cellular suspension was obtained by blood, spleen, and bone marrow and analyzed by flow cytometry.

### LPS-Induced Inflammation

**Figure 1:**
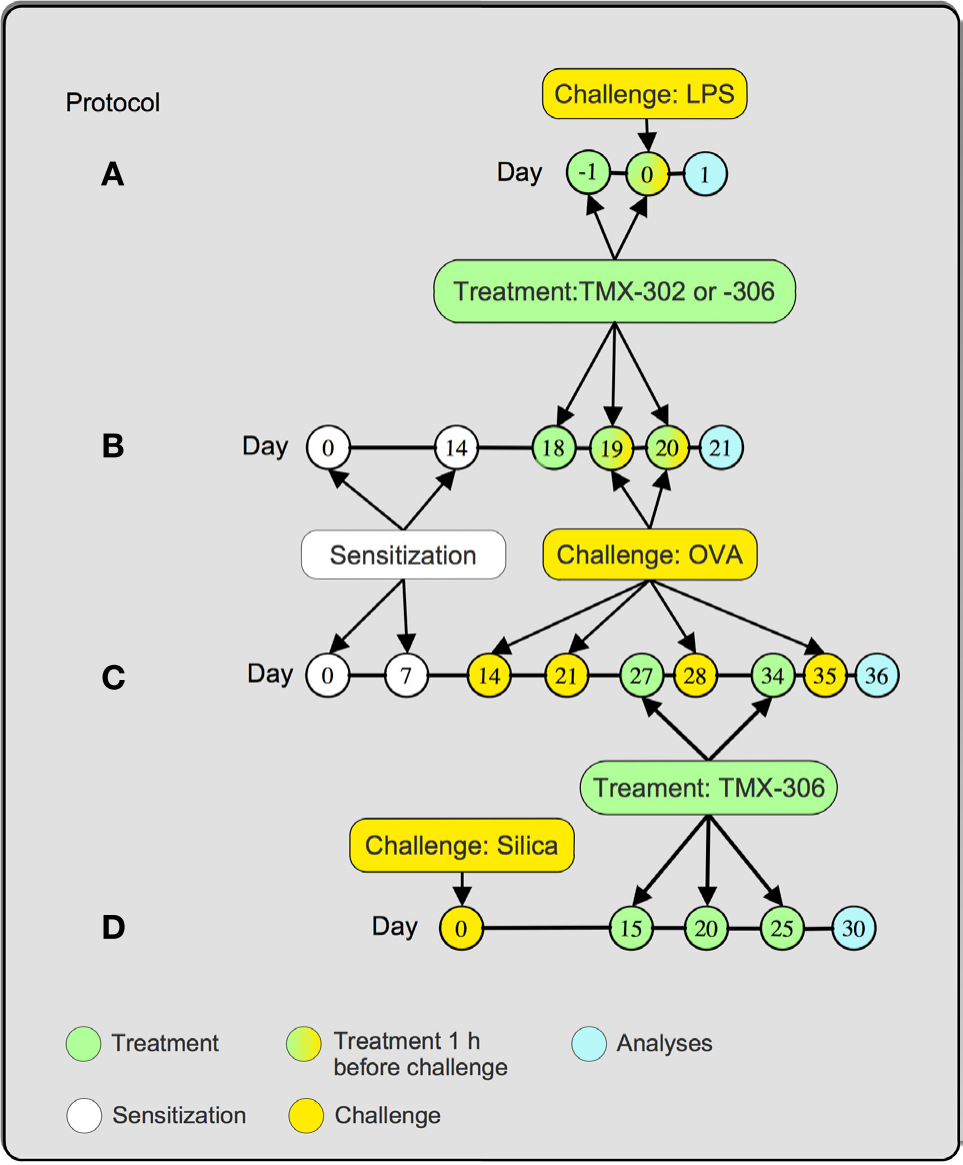
**Treatment protocols**. Mice were treated 1 and 24 h before LPS (25 μg/25 μL), and analyses were made 24 h after provocation **(A)**. Mice were sensitized at days 0 and 14 and subjected to two consecutive daily injections of OVA (25 μg/25 μL) at days 19 and 20 postsensitization. TMX-302 was given subcutaneously (500 nmoles/mouse) or intranasally (65 nmoles/mouse), 1 and 24 h before OVA. TMX-306 was aerosolized (2 and 6 mg/mL, 30 min) under the same conditions. Analyses occurred 24 h after the last challenge **(B)**. Mice were sensitized at days 0 and 7 and subjected to a series of four provocations with OVA (50 μg/25 μL) at days 14, 21, 28, and 35 postsensitization. TMX-306 (70 nmoles/mouse) was intranasally injected at days 27 and 34 **(C)**. Mice were challenged with crystalline silica particles at day 0 and subjected to three treatments with TMX-306 (70 nmoles/mouse) at days 15, 20, and 25 **(D)**. Analyses were performed at day 30 post-silica.

A/J mice were exposed to a single dose of LPS (25 μg/mouse) or phosphate buffered solution (PBS) by intranasal route. The analyses were performed 24 h after stimulation. Treatment with TMX-302 (500 nmoles/mouse) was performed subcutaneously, 1 and 24 h prior to LPS exposure (Figure 1A).

### Ovalbumin-Induced Inflammation

A/J mice were sensitized, subcutaneously, with 50 μg of OVA and 5 mg of aluminum hydroxide dissolved in 0.2 mL PBS. Two protocols to induce the allergic response were used. For the short-term protocol (Figure 1B), mice were sensitized at day 0, boosted on day 14, and then exposed to intranasal OVA (25 μg/mouse), or sterile PBS, at days 19 and 20 (33). Treated animals received TMX-302 either by subcutaneous (500 nmoles/mouse) or intranasal route (65 nmoles/mouse), 1 and 24 h before allergen challenge. In another set of experiments, mice were exposed to aerosol of TMX-306 (2 and 6 mg/mL) also following protocol B (Figure 1B). For the interventional treatment, mice were sensitized at day 0, boosted at day 7, and then challenged with OVA (50 μg/mouse), or PBS, days 14, 21, 28, and 35 postsensitization (4). Treated animals received intranasal TMX-306 (70 nmoles/mouse) or oral dexamethasone (1 mg/kg), at days 26 and 33 postsensitization. In both protocols, the analyses were performed 24 h after the last OVA challenge (Figure 1C).

### Silica-Induced Chronic Inflammation

Swiss Webster mice were exposed to crystalline silica particles (10 mg/mouse) (size 0.5–10 μm) or sterile PBS as control (1). The interventional treatment with TMX-306 (70 nmoles/mouse) was given at days 15, 20, and 25, and the analyses performed at day 30 after silica instillation (Figure 1D).

### Invasive Assessment of Respiratory Mechanics

Mice were anesthetized with nembutal (60 mg/kg), and neuromuscular activity was blocked with bromide pancuronium (1 mg/kg). Lung resistance (cmH_2_O s/mL) and elastance (mL/cmH_2_O) were assessed in tracheostomized and mechanically ventilated mice using a FinePointe R/C Buxco Platform (Buxco^®^ Electronics, Sharon, CT, USA) (1).

### Bronchoalveolar Lavage

Airways were lavaged by a polyethylene cannula, inserted into the trachea, with two consecutive instillations of 0.75 mL of PBS containing 10 mM of EDTA. Bronchoalveolar Lavage (BAL) was centrifuged at 300 × *g* for 10 min at 4°C, and the cell pellet was resuspended in 0.25 mL of PBS for leukocyte enumeration. Total cells were counted in Neubauer chamber by means of light microscopy, after dilution of samples in Turk solution. The differential analysis was performed in cytocentrifuged smears stained for identification of mononuclear cells, neutrophils, and eosinophils by May-Grunwald-Giemsa under an oil immersion objective and light microscope (BX51, Olympus) (34).

### Histology

The left lung was removed, fixed in Milloning buffer solution (pH 7.4) with 4% paraformaldehyde to preserve pulmonary architecture. Briefly, samples were embedded in paraffin (Sigma-Aldrich), and 4 μm-thick sections were cut and stained with hematoxylin and eosin for quantification of granuloma area, Picrosirius for collagen fibers and Sirius Red (pH 10.2) for neutrophils and eosinophils counted in the parenchyma and in peribronchiolar area, respectively. Slides were scanned with 3DHISTECH–Pannoramic MIDI whole slide scanner (capture with a 20× objective lens) and the resulting images analyzed with CaseViewer 3.3, Pannoramic Viewer 1.15.4, and HistoQuant softwares (3DHISTECH). Silica crystals were analyzed, in 15 independent fields, with a light microscope (Olympus BX50) equipped with polarizing attachment for detecting birefringent particles and Image-Pro Plus Version 4.

### Immunohistochemistry

Left lung samples were examined for immunohistochemical localization of TGF-β using paraffin-embedded sections. Primary anti-TGF-β1/2/3 (sc-7892) was obtained from Santa Cruz Biotechnology (Dallas, TX, USA). Secondary antibody HAF008 was conjugated with horseradish peroxidase (HRP) and obtained from R&D Systems (Minneapolis, MN, USA). In negative controls, primary antibody was omitted, and tissues were incubated with antibody diluent only. To improve visualization of the primary label, slides were counterstained with Mayer’s Hematoxylin (Lillie’s modification) as previously described (1). The slides were scanned with 3DHISTECH–Pannoramic MIDI and quantified as previously reported (1).

### Cytokine Quantification

Murine TNF-α, MIP-1α/CXCL-3, MIP-2/CXCL-2, IL-6, and eotaxin-2 levels were measured in the right lung tissue samples, which were homogenized in PBS containing 0.05% Triton X-100 and a protease inhibitor cocktail (Hoffmann-La Roche, Basel, Switzerland). Samples were quantified using commercially available ELISA kits (DuoSet system, R&D Systems, Minneapolis, MN, USA), according to the manufacturer’s instructions. The results were expressed as picograms of cytokine per right lung.

### Protein Quantification

Total protein levels were measured by Bradford technique. Right lung tissue samples were homogenized in PBS 1 mL with Triton X-100 (0.1%), containing protease inhibitor COMPLETE (Hoffmann-La Roche Ltd., Switzerland). The results were expressed as micrograms of protein per right lung.

### Statistical Analysis

Statistical analyzes were performed using GraphPad Prism Software, version 5.0 (USA). For *in vitro* experiments on human PBMCs, the analyses were performed with repeated measures two-way ANOVA followed by Tukey’s multiple comparison. For *in vivo* experiments, the analyses were done with one-way ANOVA followed by the Student–Newman–Keuls test or two-way ANOVA with *post hoc* Bonferroni correction. Statistical differences were considered significant if *p* values were less than 0.05 (two-tailed tests).

## Results

### PEGylated TLR7 Partial Agonists Activity of Human Leukocytes

**Figure 2:**
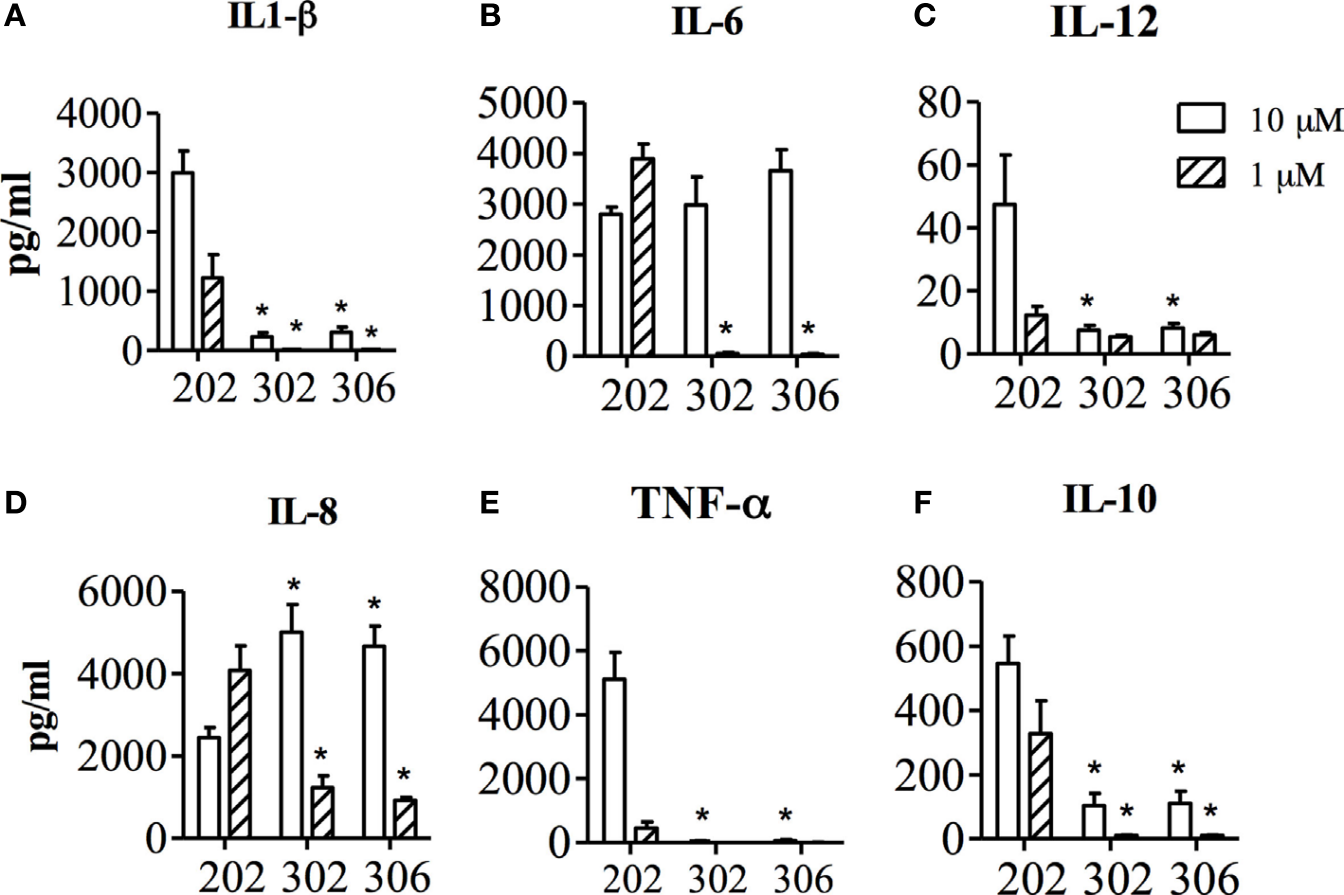
Concentration of IL1-β **(A)**, IL-6 **(B)**, IL-12 **(C)**, IL-8 **(D)**, TNF-α **(E)** and IL-10 **(F)** in the supernatant of human PBMCs, after 24 h stimulation with TMX-202, TMX-302 or TMX-306 at 1 or 10 μM. Data are presented as mean ± SEM from *n* = 6.

**Figure 3:**
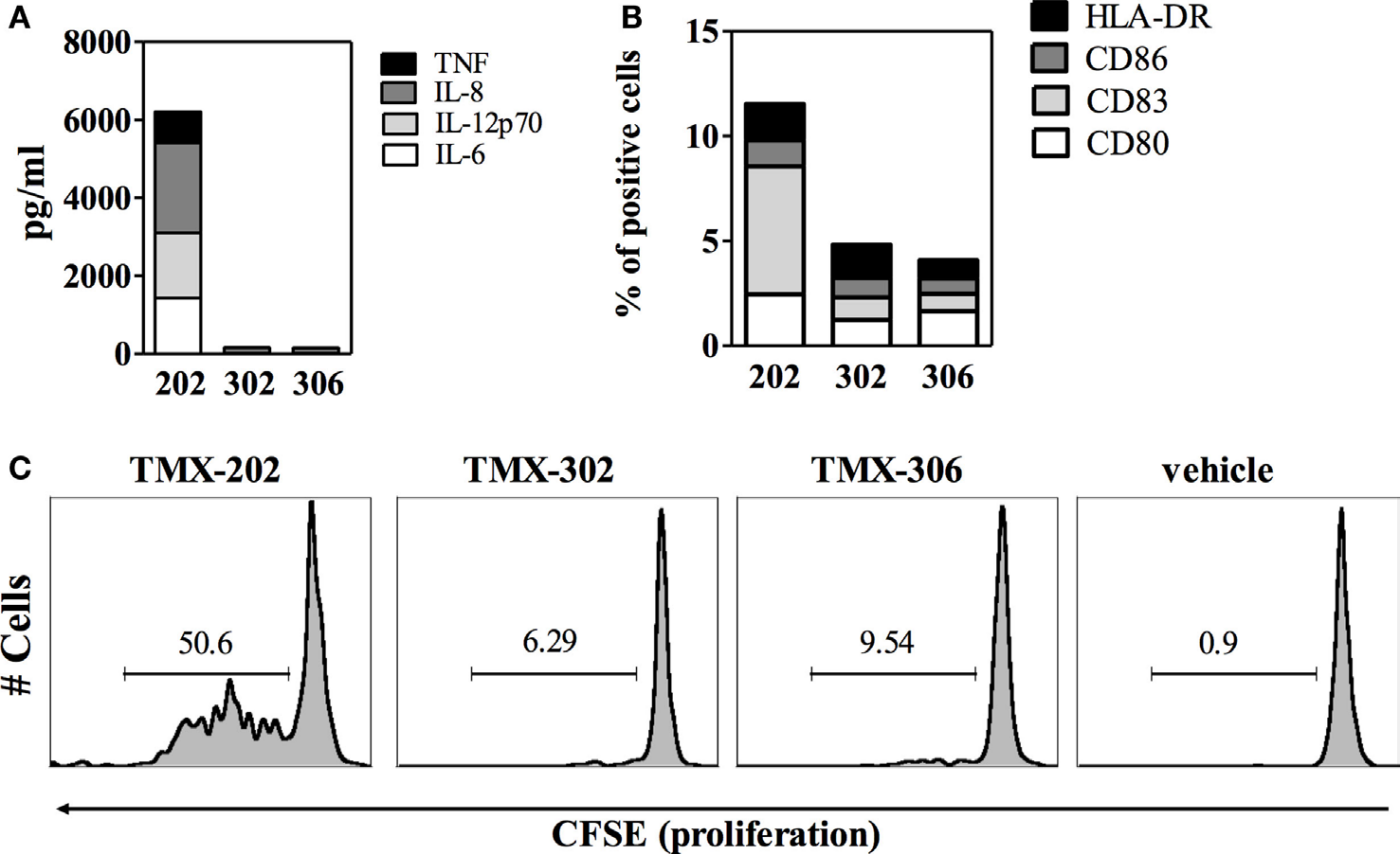
**Effect of PEGylated TLR7-agonists on maturation of mo-DCs or proliferation of B cells**. **(A)** Concentration of the indicated inflammatory cytokines in the supernatant of mo-DCs (*n* = 6), after 24 h stimulation with TMX-202, TMX-302, or TMX-306 at 10 μM. Data are presented as mean values. **(B)** Frequency of mo-DCs expressing the maturation markers CD80, CD83, CD86, and HLA-DR, after 24 h stimulation with TMX-202, TMX-302, or TMX-306 at 10 μM. Data are presented as mean values of three independent experiments. Dotted lines in **(A,B)** represent the amount of cytokines or expression of maturation markers in the absence of TLR7 stimulation. **(C)** B cell proliferation measured by CFSE dilutions after 4 days in culture with TMX-202, TMX-302, or TMX-306 at 10 μM. One representative plot out of three experiments performed with cells from different donors is shown.

The effects of the two 1V136 PEGylated derivatives, TMX-302 and TMX-306, on human PBMCs and on the maturation of mo-DCs, were assessed in cells from healthy donors. *In vitro*, none of the PEGylated compounds resulted to be toxic on PBMCs; with no relevant apoptosis induced after over night exposure to TMX-302 or TMX-306 (10 μM, data not shown). Both TMX-302 and TMX-306 induced a minimal cytokine production as compared to the TLR7 full agonist TMX-202 at 1 μM. When the compounds were used at high concentration (10 μM), we observed production only of IL–6 and IL–8 that was comparable to the one observed using TMX-202 (Figure 2). None of the tested PEGylated TLR7 partial agonists induced relevant cytokine production by ­mo-DCs (Figure 3A) or mo-DCs maturation (Figure 3B). Moreover, cytofluorimetric analysis on human PBMCs labeled with CFSE and incubated with the different compounds showed that the PEGylated partial agonists do not induce B cell proliferation (Figure 3C).

### Effect of TMX-302 on LPS-Induced Inflammation and Airway Hyper-Reactivity

**Figure 4:**
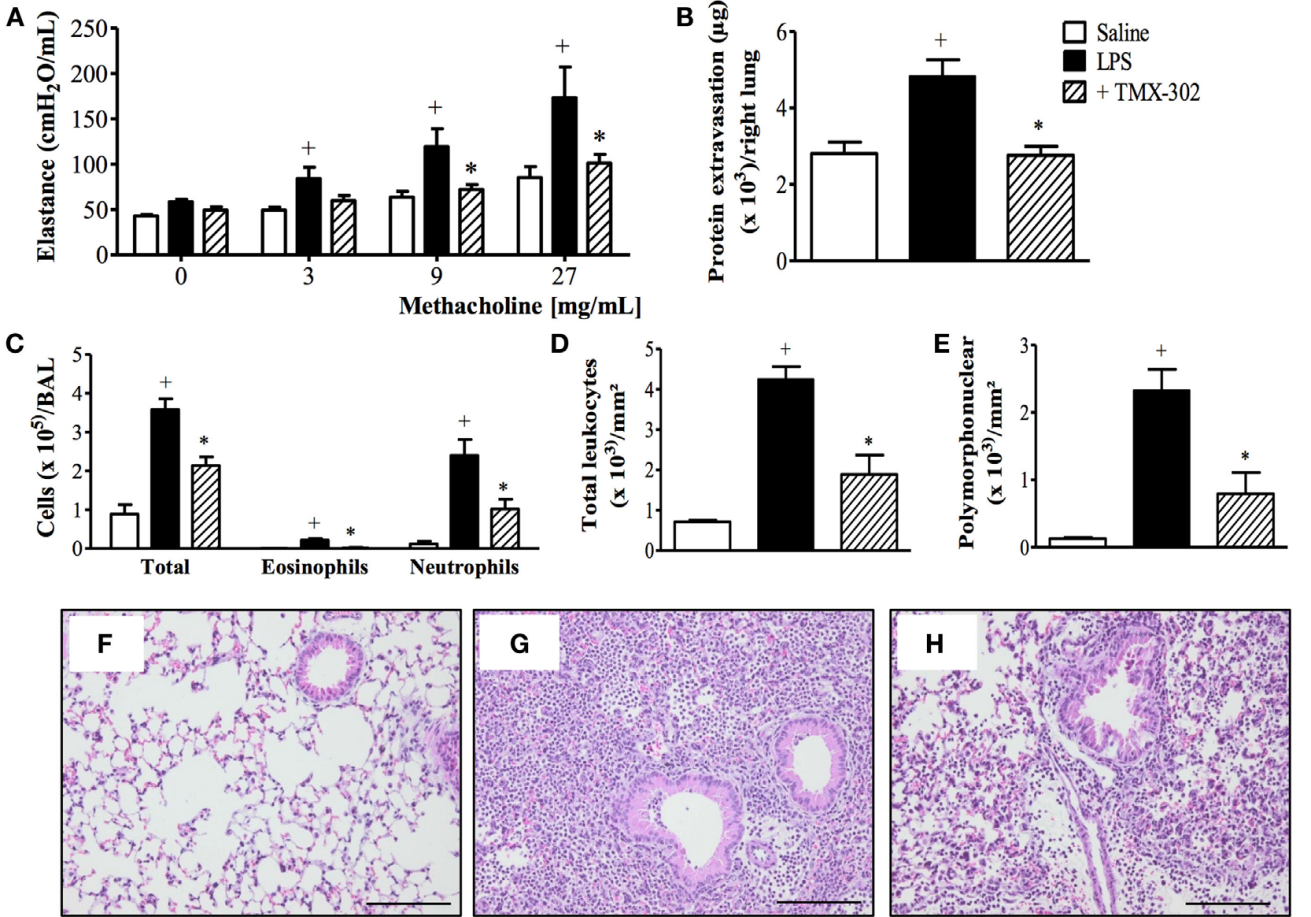
**Effect of subcutaneous treatment with TMX-302 on LPS-induced inflammation in the lung of mice**. **(A)** Lung elastance; **(B)** protein exudation; **(C)** leukocytes in BAL; **(D)** total leukocytes; and **(E)** polymorphonuclear in the lung tissue. Histological sections of lungs from animals instilled with **(F)** PBS, **(G)** LPS (25 μg/mouse), and **(H)** LPS and treated with TMX-302 (500 nmoles/mouse). Slides were stained with H&E. Scale bar = 200 μm. All the analyses were made 24 h after LPS stimulation. Values represent mean ± SEM from at least six animals. ^+^*p* < 0.05 as compared to PBS-challenged group; *p < 0.05 as compared to LPS-challenged group.

Subcutaneous pre-treatment with TMX-302 (500 nmoles/mouse given twice), 1 and 24 h before LPS (protocol A, Figure 1), reduced the increased lung elastance response noted in mice challenged with LPS (Figure 4A). As expected, LPS also caused protein extravasation (Figure 4B) and augmentation in total leukocyte levels, as indicated by enumeration of these cells in BAL effluents (Figure 4C) and lung histological sections (Figures 4D,G), in comparison to the respective negative controls (Figures 4C,F). Neutrophils were the predominant leukocyte subtype found in the bronchoalveolar space (Figure 4C) and lung parenchyma (Figures 4E,G). All these changes were significantly inhibited by the subcutaneous pre-treatment with TMX-302 (Figures 4B–E,H). Quantification of pro-inflammatory cytokines and chemokines in lung homogenates in response to LPS revealed increased levels of TNF-α, MIP-1α, and IL-6, all of which appeared inhibited by TMX-302 though, in case of IL-6, the 40% blockade was not statistically significant (Table 1).

**Table 1:** **Effect of TMX-302 on cytokine/chemokine generation in the lung tissue of LPS-stimulated mice**.

Cytokine (pg/lung tissue)	PBS	LPS	LPS + TMX-302
TNF-α	97.5 ± 8.7	290.2 ± 49.2^+^	178.9 ± 29.1*
MIP-1α	314.5 ± 48.6	1276.1 ± 277.7^+^	816.9 ± 120.6*
IL-6	280.2 ± 57.7	1131.1 ± 285.0^+^	777.6 ± 122.2

*TMX-302 (500 nmoles/mouse) was given 1 h before LPS (25 μg), and the analyses were performed 24 h after LPS. Values represent the mean ± SEM from at least six animals*.

**^+^p* < 0.05 vs. PBS-challenged group*.

*p < 0.05 vs. LPS-challenged group.

### Effect of TMX-302 on Allergen-Induced Inflammation and Airway Hyper-Reactivity

**Figure 5:**
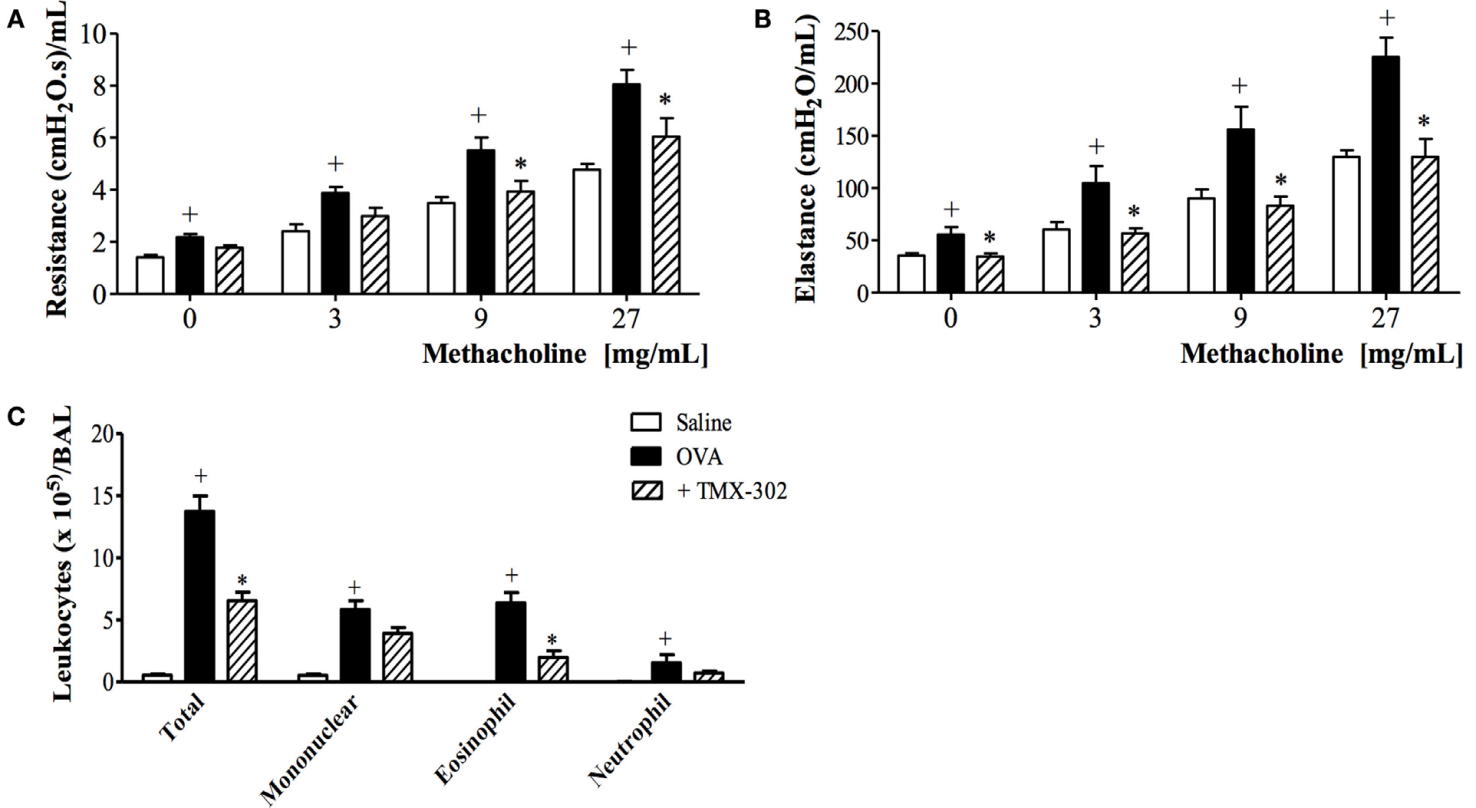
**Effect of subcutaneous treatment with TMX-302 on OVA-induced inflammation in the lung of mice**. Lung function: **(A)** resistance and **(B)** elastance **(C)** total leukocytes in BAL. Animals were sensitized on days 0 and 7 and then challenged with OVA (25 μg/mouse) or PBS, on days 19 and 20. Treatment with TMX-302 (500 nmoles/mouse, subcutaneous) was given 1 h before each OVA challenge, and analyses were performed 24 h after the last stimulation. Values represent mean ± SEM from at least six animals. ^+^*p* < 0.05 as compared to PBS-challenged group; *p < 0.05 as compared to OVA-challenged group.

Confirming previous reports (33), OVA intranasal challenge of sensitized mice exacerbated both airway resistance (Figure 5A) and lung elastance responses (Figure 5B) to inhaled methacholine (3–27 mg/mL). Increased levels of leukocytes, mainly eosinophils, were detected in the BAL fluid (Figure 5C) as compared to control mice challenged with PBS. The same figures show that the pre-treatment with TMX-302 (500 nmoles/mouse, subcutaneous) (protocol B, Figure 1) prevented allergen-induced airway hyper-reactivity (AHR) and eosinophilic leukocyte accumulation (Figures 5A–C).

**Figure 6:**
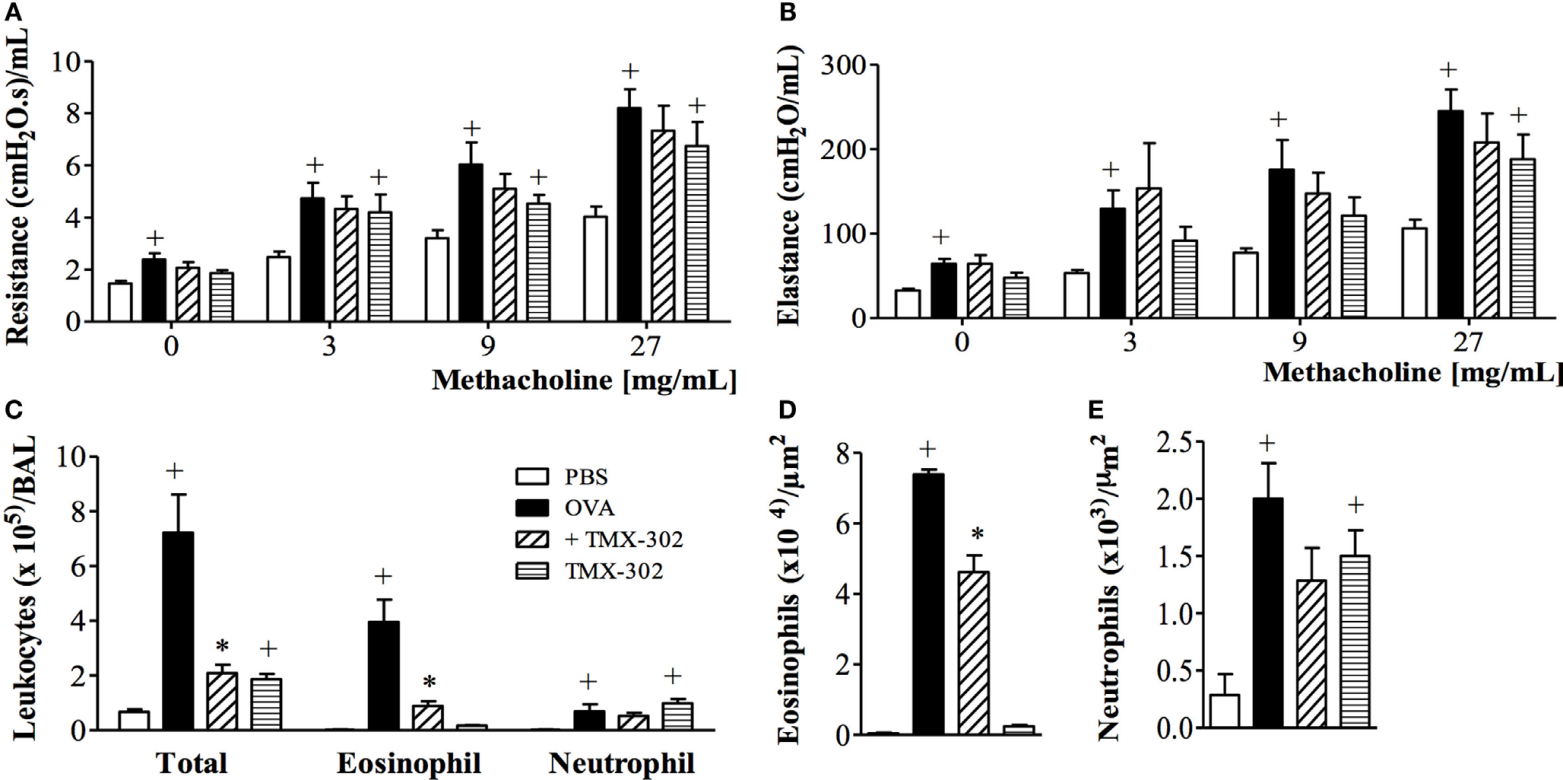
**Effect of intranasal treatment with TMX-302 on OVA-induced inflammation in the lung of mice**. Lung function: **(A)** resistance; **(B)** elastance, **(C)** total leukocytes in BAL, **(D)** tissue eosinophil numbers and **(E)** tissue neutrophil numbers. Animals were sensitized on days 0 and 7 and then challenged with OVA (25 μg/mouse) or PBS on days 19 and 20. Treatment with TMX-302 (65 nmoles/mouse, intranasal) was given 1 h before each OVA challenge, and analyses were performed 24 h after the last stimulation. Values represent mean ± SEM from at least six animals. ^+^*p* < 0.05 as compared to PBS-challenged group; *p < 0.05 as compared to OVA-challenged group.

When the systemic prophylactic was replaced by the local prophylactic treatment with TMX-302 (65 nmoles/mouse, intranasal instillation) (protocol B, Figure 1), no more protective effect was seen for allergen-induced increased airway resistance (Figure 6A) and lung elastance (Figure 6B) in response to methacholine. Moreover, TMX-302 itself caused AHR in naive mice (Figures 6A,B). A significant blockade of the OVA-induced eosinophilic, but not neutrophilic infiltration, was apparent following TMX-302, as observed in BAL samples (Figure 6C) and lung tissue samples (Figures 6D,E). Moreover, the nasal instillation of TMX-302 (65 nmoles/mouse) in naive mice led to neutrophil accumulations in the BAL fluid (Figure 6C) and, particularly, in the lung parenchyma (Figure 6E). As shown in Table 2, the intranasal instillation of TMX-302, despite inhibiting OVA-induced TNF-α and MIP-2, clearly up-regulated OVA-induced MIP-1-α, and caused itself significant elevation of MIP-1α, MIP-2, and TNF-α levels in lung homogenates.

**Table 2:** **Effect of TMX-302 on cytokine/chemokine generation in the lung tissue of allergen-stimulated mice**.

Cytokine (pg/lung tissue)	PBS	OVA	OVA + TMX-302	TMX-302
MIP-1-α	117.2 ± 76.4	937.5 ± 169^+^	1672.7 ± 295.1*	605.4 ± 84.9^+^
MIP-2	275.6 ± 70.6	665.6 ± 66.8^+^	243.9 ± 68.7*	934.9 ± 126.8^+^
TNF-α	88.7 ± 7.6	138.9 ± 11.0^+^	110.1 ± 8.7*	132.1 ± 11.2^+^

*The analyses were performed 24 h after ovalbumin provocation, and values represent the mean ± SEM from at least six animals*.

*^+^*p* < 0.05 vs. PBS-challenge group*.

*p < 0.05 vs. OVA-challenge group.

### Effect of TMX-306 on Allergen-Induced Inflammation and Airway Hyper-Reactivity

**Figure 7:**
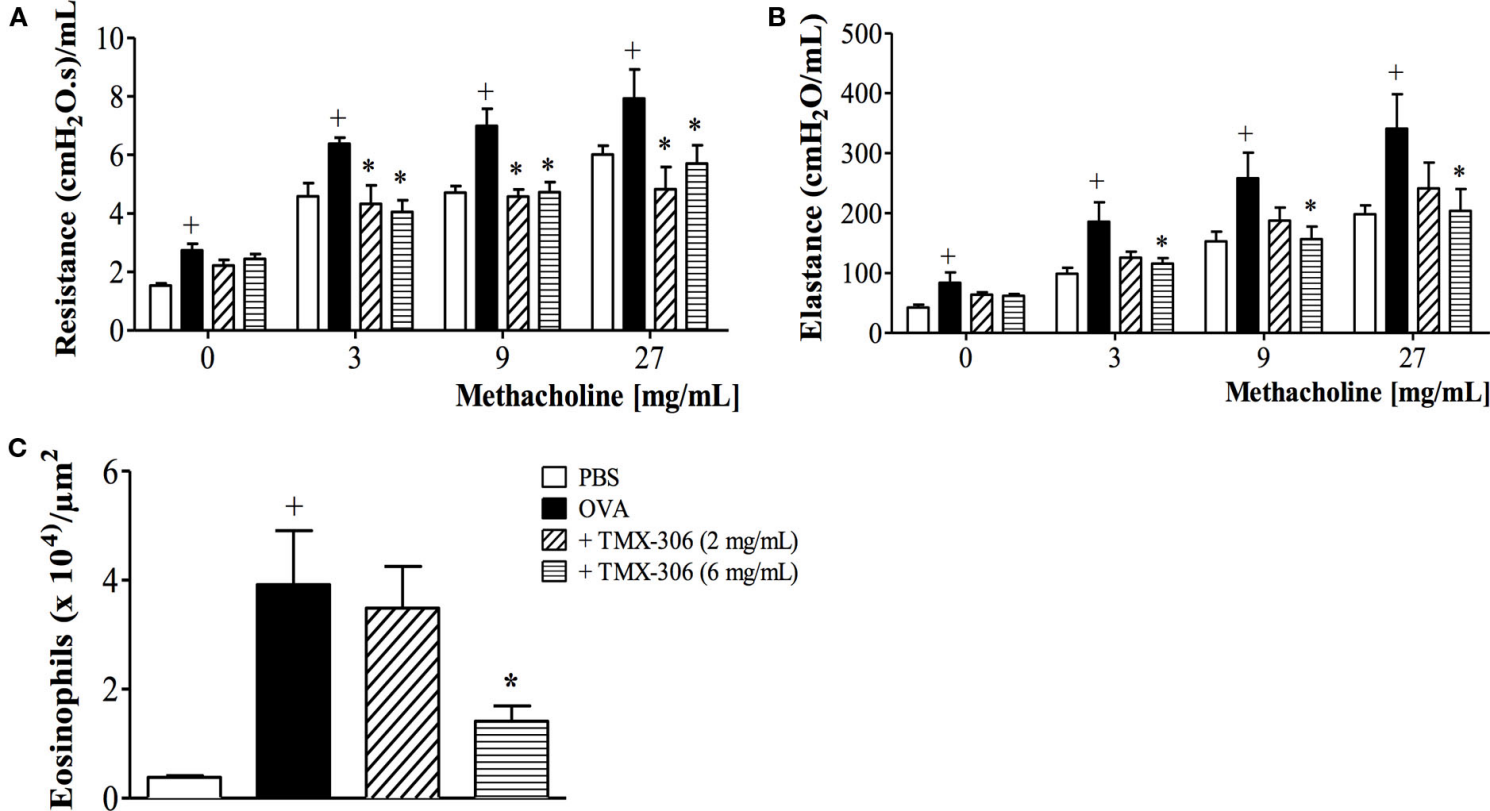
**Effect of aerosol treatment with TMX-306 on OVA-induced inflammation in the lung of mice**. Lung function: **(A)** resistance, **(B)** elastance, and **(C)** peribronchiolar eosinophil infiltration. Animals were sensitized on days 0 and 7 and then challenged with OVA (25 μg/mouse) or PBS on days 19 and 20. Treatment with TMX-306 (2 and 6 mg/mL, aerosol) was given 1 h before each OVA challenge, and analyses were performed 24 h after the last stimulation. Values represent mean ± SEM from at least six animals. ^+^*p* < 0.05 as compared to PBS-challenged group; *p < 0.05 as compared to OVA-challenged group.

Once the topical administration, through intranasal instillation of TMX-302, was ineffective upon asthmatic changes and caused adverse events, the effects of the parent compound TMX-306 were investigated. As shown in Figure 7, the prophylactic treatment with aerosolized TMX-306 (6 mg/mL) (protocol B, Figure 1) prevented allergen-induced AHR, in respect to airway resistance (Figure 7A) and lung elastance (Figure 7B), as well as the ­infiltration of eosinophils in the peribronchiolar zone (Figure 7C). When aerosolized at 2 mg/mL, TMX-306 prevented allergen-induced AHR but not eosinophilic infiltration (Figures 7A–C).

**Figure 8:**
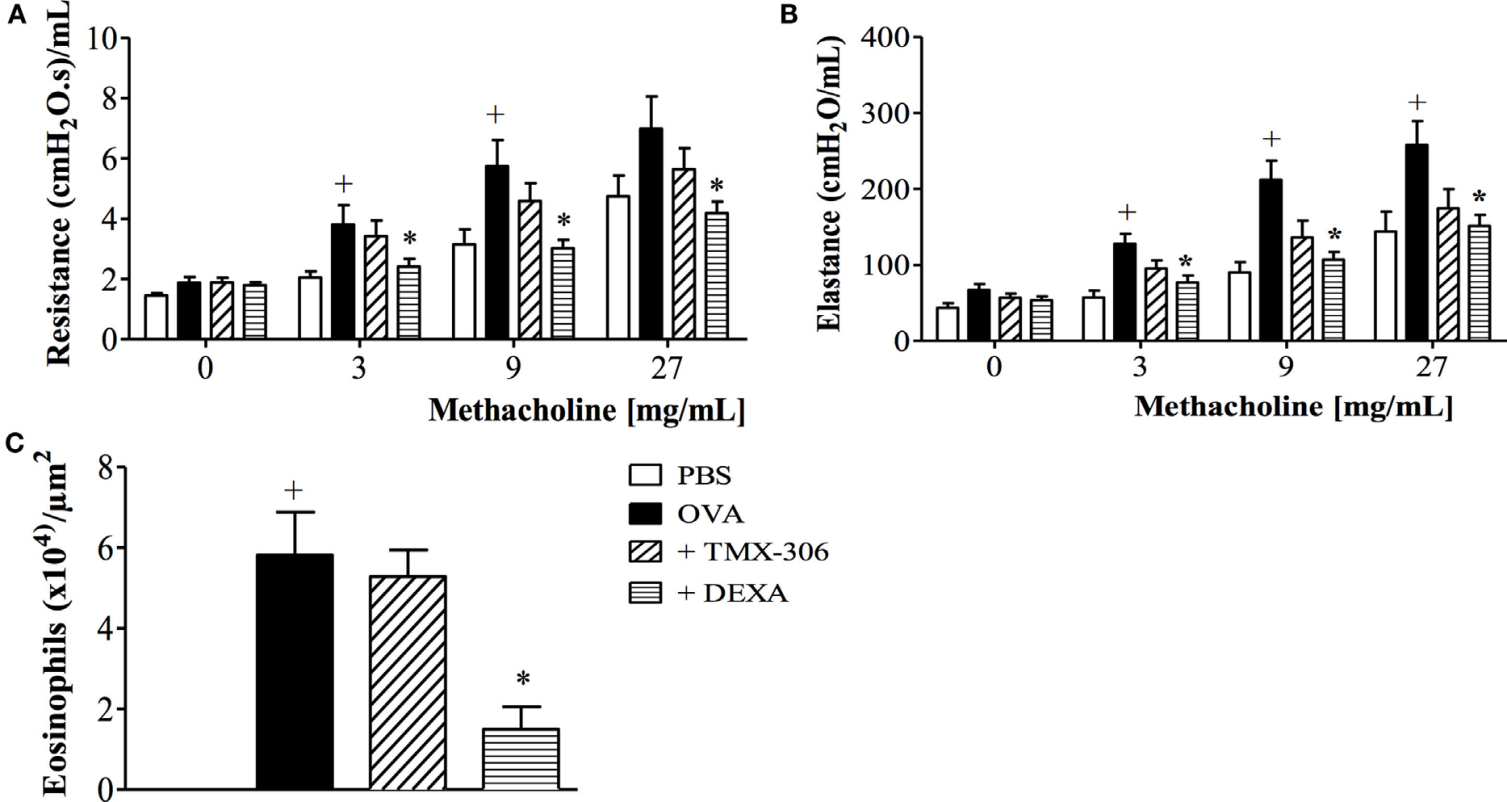
**Effect of intranasal treatment with TMX-306 on OVA-induced inflammation in the lung of mice**. Lung function: **(A)** resistance; **(B)** elastance, and **(C)** peribronchiolar eosinophil infiltration. Animals were sensitized on days 0 and 7 and then challenged with OVA (25 μg/mouse) or PBS on days 14, 21, 28, and 35. Animals were treated with TMX-306 (70 nmoles/mouse, intranasal) or dexamethasone (1 mg/Kg, oral) on days 26 and 22, 1 h before OVA challenge, and analyses performed 24 h after the last challenge. Values represent mean ± SEM from at least six animals. ^+^*p* < 0.05 as compared to PBS-challenged group; *p < 0.05 as compared to OVA-challenged group.

We next assessed the effectiveness of TMX-306 (70 nmoles/mouse, intranasal) on ongoing asthmatic changes according to the protocol C (Figure 1). Contrarily to dexamethasone, TMX-306 failed to reduce AHR (Figures 8A,B) and peribronchiolar eosinophilic infiltration (Figure 8C) caused by OVA challenge, suggesting the lack of beneficial effects for the therapeutic treatment with TMX-306 on allergen-induced pathological changes, under the conditions applied.

### Effect of TMX-306 on Silica-Induced Inflammatory, Fibrotic, and Respiratory Changes

Initially, in order to assess if the PEGylated compound TMX-306 would impact *per se* cell mobilization from the bone marrow as well as subsequent distribution in blood circulation and spleen, TMX-306 was injected intraperitoneally in mice. The results obtained indicate that this PEGylated analogue, at the dose of 200 nmoles/mouse, does not affect cell mobilization and compartmentalization in wild type mice (Figure S1 in Supplementary Material).

**Figure 9:**
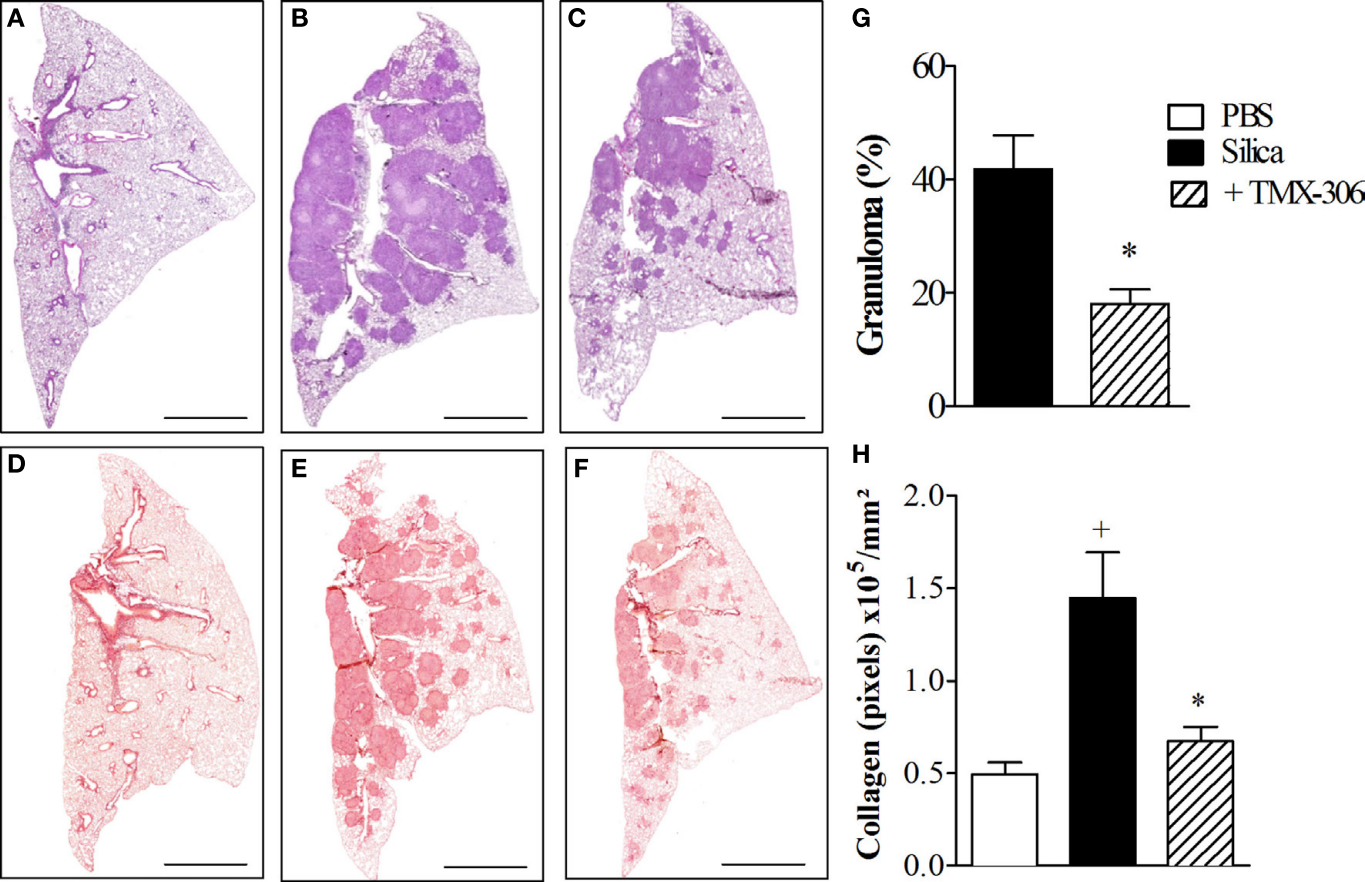
**Effect of intranasal treatment with TMX-306 on granuloma formation (upper panels) and collagen deposition (lower panels) in ­silica-challenged mice**. Histological sections of mouse lungs on day 30 after silica challenge **(B,E)** and treated with TMX-306 (70 nmoles/mouse, intranasal) **(C,F)**. Animals instilled with PBS were used as controls **(A,D)**. Animals were treated with TMX-306 (70 nmoles/mouse, intranasal) days 15, 20, and 25 post-silica. Morphometric analyzes are seen in **(G)** granuloma area and **(H)** collagen deposition. Slides were stained with H&E (upper panels) and Picrosirius red (lower panels). Scale bars, 200 μm. Values represent mean ± SEM from at least five animals. ^+^*P* < 0.05 as compared to PBS-challenged group; *P < 0.05 as compared to silica-challenged group.

The next step was the evaluation of the effect of TMX-306 on experimental silicosis, which was performed in accordance to protocol D (Figure 1) and prior investigations (1, 35). Based on the histologic analyzes of lung sections stained with H&E for assessment of granuloma (Figure 9, upper panels) and Picrosirius red for evaluation of fibrotic lesions (Figure 9, lower panels), it became clear that, compared to mice exposed to PBS (Figure 9A), those exposed to silica particles (Figure 9B) reacted with an intense granulomatous response, which occupied about 40% of the left pulmonary lobe 30 days postprovocation (Figure 9G). Moreover, a dense area of collagen fiber deposition appeared distributed in those spaces occupied by granuloma in silicotic mice (Figure 9E). Remarkably, both granuloma and fibrotic lesions caused by silica inhalation were clearly inhibited by the treatment with TMX-306 (70 nmoles/mouse), carried out at days 15, 20, and 25 after silica provocation (Figures 9G,H, respectively).

**Figure 10:**
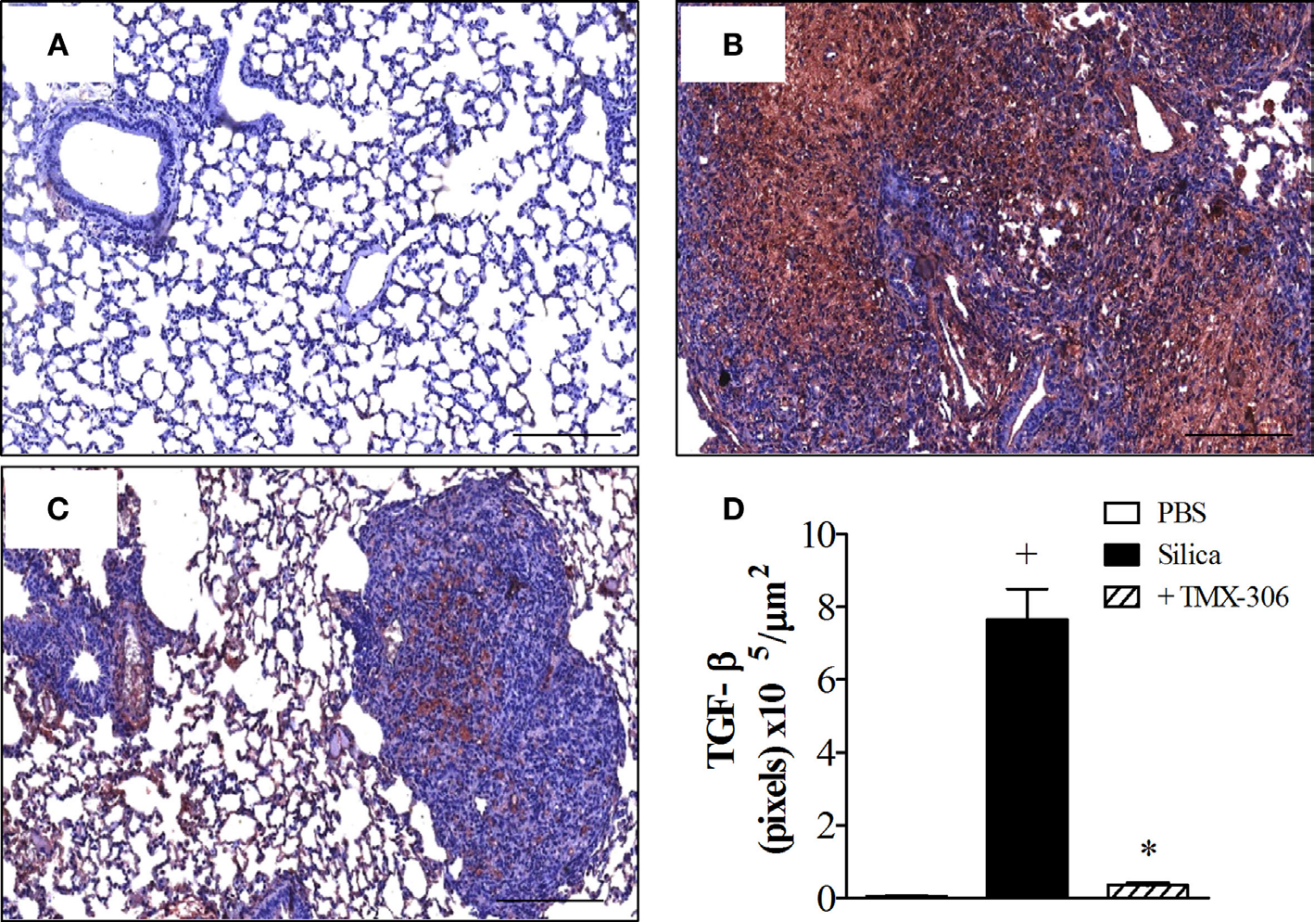
**Effect of intranasal treatment with TMX-306 on TGF-β production in the lung tissue of silica-challenged mice**. Samples were analyzed in animals instilled with PBS **(A)**, silica (10 mg/mouse) **(B)**, and silica treated with TMX-306 (70 nmoles/mouse, intranasal) **(C)** 30 days after silica challenge. Treatment with TMX-306 was performed at days 15, 20, and 25 post-silica. Quantitative analyses are seen in **(D)**. Values represent mean ± SEM from at least six animals. ^+^*P* < 0.05 as compared to PBS-challenged group; *P < 0.05 as compared to silica-challenged group.

Using immunohistochemistry technique based on anti-TGF-β staining, the quantitative assessment of expression of TGF-β under conditions of exposure to PBS, silica particles, or silica plus TMX-306 revealed significant increase in lung tissue levels of IL-TGF-β in samples recovered from mice exposed to silica particles (Figure 10B), as compared to those exposed to PBS (Figure 10A). Given through nasal instillation in the regime mentioned before, TMX-306 inhibited silica-induced production of TGFβ (Figure 10C). The quantitative data are shown in Figure 10D.

### Effect of TMX-306 on Silica Particle Diffusion in Lung Parenchyma

**Figure 11:**
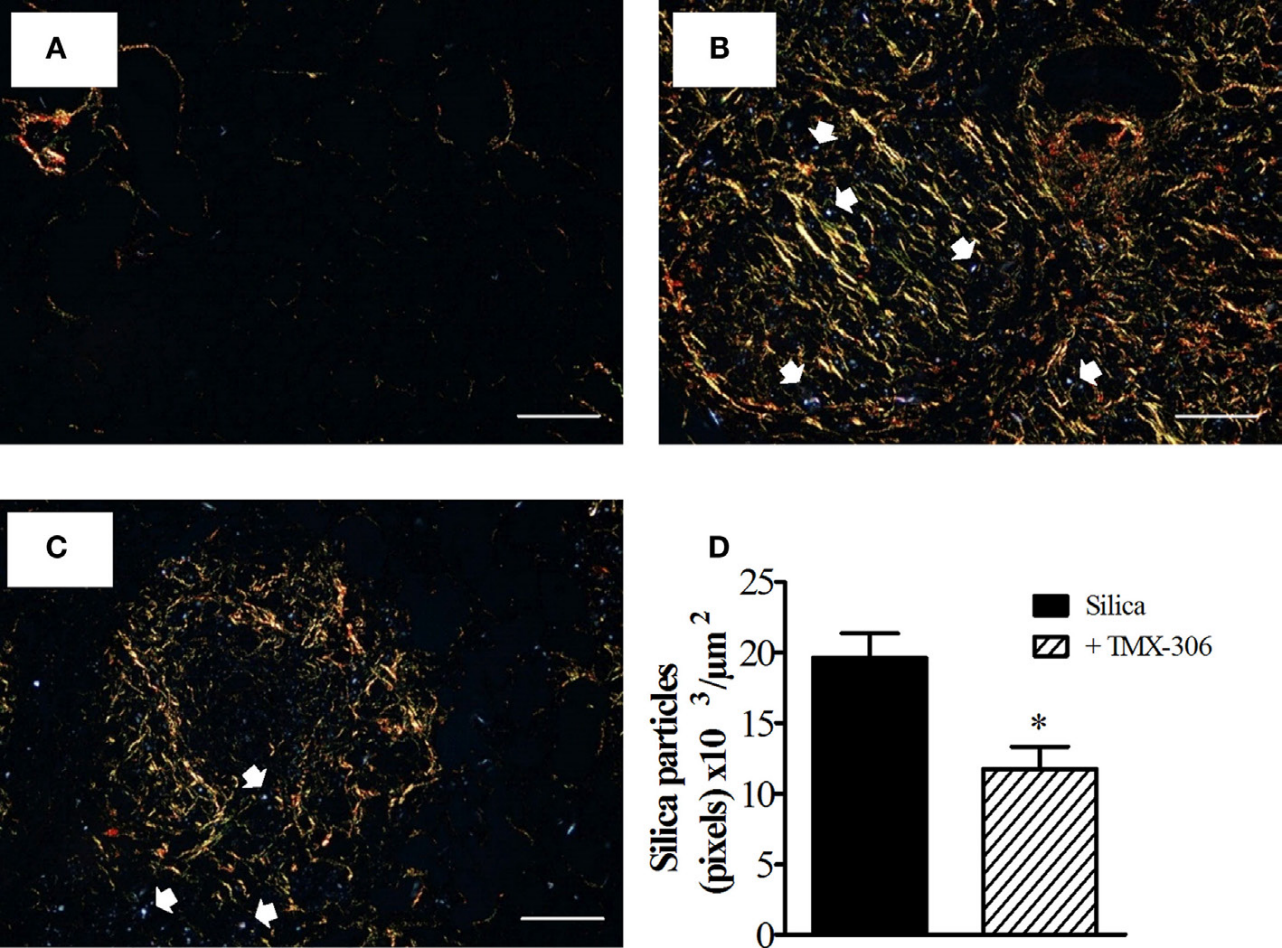
**TMX-306 reduces silica particles in lung tissue**. Quantitative evaluation of silica particles was assessed in animals instilled with PBS **(A)**, silica (10 mg/mouse) **(B)**, and silica treated with TMX-306 (70 nmoles/mouse, intranasal) **(C)**, 30 days after silica challenge. Quantitative analyses are seen in **(D)**. Picrosirius red-stained sections were evaluated by light and polarized microscopy, respectively. Arrows indicate silica particles. Scale bar, 200 μm. Values represent mean ± SEM from at least seven animals. *P < 0.05 as compared to silica-challenged group.

In this study, we used a light microscope equipped with polarizing filters when examining lung tissue sections from mice exposed to silica particles. Having lung section from mice exposed to PBS as reference (Figure 11A), our findings confirmed the presence of numerous crystals of silica in sections from mice exposed to the particles, seen as small bluish bright specks, distributed throughout lung areas mainly those occupied by granuloma (Figure 11B). The amount of silica particles present in the interstitial space appeared significantly reduced in mice treated with TMX-306 (70 nmoles/mouse) (Figure 11C). Quantitative data are shown in Figure 11D.

## Discussion

Toll-like receptors play a crucial role in sensing and responding to respirable “dangerous triggers” including allergens and ambient pollutant particles, which may lead to asthma and pneumoconiosis (13, 36). Synthetic low molecular weight TLR7 agonists, including 1V136 and others, have been shown to down-regulate immune responses during inflammatory conditions (25, 26, 37, 38). Additionally, PEGylation improves bioavailability and safety of these ligands (30, 39). The overall purpose of this study was to access the effects of 1V136 PEGylated derivatives on pulmonary inflammatory and functional changes caused by three distinct classes of pathogens, including LPS, allergen, and crystalline silica particles.

Our experiments revealed that both PEGylated derivatives TMX-302 and TMX-306 presented a marginal pro-inflammatory response, yielding a minimal production of inflammatory cytokines in human PBMCs, no mo-DC maturation or B cell proliferative responses, differently from the TLR7 full agonist used for comparison (TMX-202). In *in vivo* settings, subcutaneous pre-treatment with TMX-302 prevented LPS- and allergen-induced lung inflammation and AHR, while its topical administration failed to prevent allergen-induced AHR, and caused itself neutrophil infiltration, in parallel with cytokine and chemokine generation. Administered topically, TMX-306 prevented allergen-induced asthma changes, but did not modify them as given therapeutically. In contrast, in the silicosis model, interventional treatment with TMX-306 significantly reduced the pulmonary fibrogranulomatous response following crystallized silica particle inhalation in mice. Altogether, these studies highlight the putative value of TMX-306 in drug development for silicosis.

As candidates to anti-inflammatory therapy, TLR7 ligands should ideally be able to push the innate system to a state of tolerance, with minimal pro-inflammatory effects. Actually, the safe therapeutic use of TLR7 agonists has been proved to be a difficult task because of the cytokine release syndrome and pharmacokinetic limitations (16). Prior investigations have demonstrated that the conjugation of these ligands to polysaccharides, serum albumin or PEG widely improved their pharmacokinetics and pharmacodynamics properties (30, 39, 40). Accordingly, in our experiments assessing cytokine production by human PBMCs *in vitro*, the PEGylated compounds TMX-302 and TMX-306, at 1 μM, were clearly less active than the reference compound TMX-202, producing marginal amounts of IL-1β, IL-6, IL-12p70, IL-8, or TNF. TMX-302 and TMX-306 at 10 μM promoted only IL-6 and IL-8 release from human PBMCs, but failed to induce pro-inflammatory cytokine release by mo-DC and their maturation as well as B lymphocyte proliferation, which are hallmarks of TLR7 activation (41), supporting the interpretation that the two PEGylated 1V136 derivatives are indeed suitable molecules for further *in vivo* investigations.

The immune-regulatory effects of TLR ligands, in more ample sense, are heterogeneous and complex. For instance, inhalation of the TLR4 agonist LPS exacerbates silica-induced fibrogranulomatous pulmonary dysfunction in mice (42), but can attenuate ongoing asthmatic changes following long-term exposure of mice to allergen challenge (43). While investigating the therapeutic potential of TMX-302 and TMX-306, we have explored well-established murine models of acute lung injury (ALI) (44), asthma (4, 33, 34), and silicosis (1, 35). ALI is a severe clinical problem associated with elevated rates of morbidity and mortality worldwide (45). Triggered by LPS, a component of the cell wall of gram-negative bacteria, ALI is marked by pulmonary neutrophilic leukocyte infiltration, disruption of the endothelial and alveolar epithelial barrier, lung edema, and severe hypoxemia (45). We found that under conditions of intranasal instillation of LPS, the systemic pre-treatment with TMX-302 (500 nmoles/mouse), given subcutaneously 24 and 1 h before provocation, clearly inhibited the lung inflammatory changes, including the massive leukocyte accumulation in the bronchoalveolar space and lung parenchyma, plasma leakage and AHR-noted 24 h postchallenge. The protective effect of TMX-302 concerning leukocyte changes and respiratory function might be explained by the blockade of pro-inflammatory cytokine production, including TNF-α, IL-6 and MIP-1α, as attested by measurements done in lung tissue samples. Recent studies emphasize the involvement of the adapter molecule MyD88 in the LPS-TLR4 signaling pathway followed by activation of NF-kappa B in ALI (46, 47), though this is still a debatable issue (48). However, since MyD88 is a pivotal adapter to all TLRs, except TLR3, the possibility does exist that TMX-302 is acting here through induction of a tolerogenic mechanism accounted for by induction of cross-desensitization between TLR7 and TLR4 signaling pathways.

Differently from TLR4, TLR7 selectively detects viral RNA, leading to activation of T-helper cell (Th1) immune response and viral clearance. Several pieces of evidence suggest that TLR activation are protective against T-helper cell (Th2)-mediated diseases, such as asthma, possibly by interfering with the Th1 versus Th2 immune balance (15, 16, 23). Additionally, activation of TLR7 expressed on CD4^+^ T cells and airway nerves can lead to anergy (49) and respiratory smooth muscle relaxation (24), respectively. TLR7 has also raised interest in asthma because respiratory viruses are a major cause of exacerbations. Notably, virus clearance depends on TLR-mediated Th1 response, which is down-regulated in the asthma Th2 microenvironment (50). We observed here that the systemic pre-treatment with TMX-302, given subcutaneously, prevented allergen-induced eosinophilic inflammatory infiltration and AHR in a short-term murine model of asthma. Nevertheless, the mucosal administration of TMX-302 (65 nmoles/mouse, intranasal instillation), 24 and 1 h before provocation, failed to prevent allergen-induced AHR though inhibiting the accumulation of eosinophils in the bronchoalveolar space and tissue samples. Actually, TMX-302 itself induced a significant increase in the levels of peribronchial neutrophils, in parallel with significant increase in the lung tissue production of MIP-1α, MIP-2, and TNFα, suggesting that caution in its use is required. We then decided to assess the effect of the analogue TMX-306, which is a molecular simplification of TMX-302 resulting from deletion of the triazol ring. It is relevant to mention that the intraperitoneal injection of 200 nmoles TMX-306 did not cause statistically significant changes in the number of monocytes, macrophages, neutrophils, T cells, or B cells in the bone marrow, spleen, and blood circulation in mice (Figure S1 in Supplementary Material). TMX-306 (500 nmoles/kg, ­subcutaneous) inhibited LPS-induced AHR as well as neutrophilic infiltration in samples of bronchoalvelar lavage (data not shown). Because the lungs provide a suitable route for aerosol delivery, we also tested the prophylactic treatment with aerosolized TMX-306 (6 mg/mL), which turned out to be effective in this model, preventing both eosinophilic infiltration and AHR triggered by allergen challenge. However, using a long-term model of asthma, TMX-306 (70 nmoles/mouse, intranasal) failed to modify the ongoing pathological changes triggered by allergen provocation, whereas the glucocorticoid agent dexamethasone was shown to be clearly active. These findings might suggest that, despite inhibiting LPS- and allergen-induced lung inflammation and AHR as given prophylactically, TMX-306 would not be as effective in modifying already established asthmatic changes following therapeutic administration.

In this study, we also investigated whether or not the pharmacological modulation of TLR7 with TMX-306 could be used to reduce silicosis. Remarkably contrasting with the lack of efficacy of the interventional TMX-306 treatment on experimental asthma, the therapeutic intranasal administration of this compound clearly attenuated lung inflammation, granuloma formation, fibrosis, and the functional respiratory changes noted in response to silica particles. Current thinking is that the pathogenesis of silicosis is largely attributed to the direct damage by silica particles to alternatively activated alveolar macrophages and DCs, engaged in the recognition, uptake, and clearance of silica particles and other environmental particulate matters that traffic in the lung (14, 51). When this barrier is broken, free silica crystals accumulate in the interstitial space and are taken up by M1 macrophages, which play a crucial role in promoting a state of pulmonary inflammation that evolves to granuloma formation and overlaps with fibrogenic areas in humans and animal models (14). Indeed, stronger lung inflammatory and fibrotic responses were noted in mice genetically deficient in macrophage receptors with collagenous structure (MARCO), a scavenger receptor deeply involved in the sense and uptake of crystalline silica by alveolar macrophages (52). This result gives support to the interpretation that M2 alveolar macrophages account for by the clearance while M1 interstitial macrophages drive the silica-induced inflammatory response (14). Remarkably, scavenger receptor class A type I/II (CD204) null mice fail to develop fibrosis following silica inhalation, in spite of keeping inflammation, suggesting that the CD204 are crucial to the development of fibrosis and resolution of inflammation (12).

In our experimental conditions, mice exposed to a single intranasal instillation of 10 mg of crystalized silica particles reacted with a progressive lung granulomatous response, which reach about 40% of the lung area 30 days postchallenge, as evidenced by scanned histopathological images of lung sections. In parallel, we found a marked increase in the levels of collagen fiber deposition, evidenced by Picrosirius red staining, which appeared densely distributed throughout areas occupied by granuloma, as previously reported (1, 35). These changes were clearly reversed following intranasal instillation of TMX-306, given at days 15, 20, and 25 post-silica. Furthermore, the extension of lung area occupied by granuloma appeared reduced in about 60% whereas a reduction of 95% was noted in the amount of deposited collagen. TMX-306 also almost abolished the levels of the pro-fibrotic TGF-β generated in response to silica exposure. Crystalline silica particles diffract light and appear as bright bluish specks against the dark tissue background, and can be seen under light microscope equipped with polarizing filters (53). Using this technique, we could detect reduction of about 40% in the number of crystals of silica dispersed in the lung interstitial space, strongly suggesting that TMX-306, by reducing areas of granuloma and fibrosis, is probably favoring silica particle mobility and clearance from the lung through lymphatic draining. In fact, prior investigations have demonstrated that silica particles can be drained by the lymphatic system to the lymph nodes, particularly under conditions of effective anti-silicosis therapy (1).

Apart from the distinct impact on the silica-induced fibrotic response, our findings are very much in line with those ones reported by Re and collaborators (54). These authors found a significant reduction of lung inflammation and granuloma formation in MyD88-KO mice after silica, giving support to the interpretation that MyD88-related innate immunity is crucial in silicosis. In addition, like ours, their results showed a robust reduction in the fibrotic response to silica in granuloma areas, with the difference that increased levels of silica-induced collagen deposition were detected throughout the lung parenchymal area (54), suggesting that inflammatory and fibrotic responses to silica can be uncoupled, which did not happen in our experimental conditions.

We cannot exclude the possibility that the PEGylated compounds are simply less potent than the full agonist TMX-202, without any necessary impact on their efficacy on the TLR7. In addition, contrary to TMX-202, the intravenous administration of PEGylated analogues such as TMX-302 and others (200 nmoles/kg) failed to alter the systemic baseline levels of pro-inflammatory cytokines such as TNF-α and IL-6 (data not shown). Further studies and more accurate toxicological investigations should be carried out on candidate compounds such as TMX-302 and TMX-306.

In conclusion, these findings provide a comprehensive comparison of the anti-inflammatory effectiveness of two PEGylated TLR7 partial agonists, concerning distinct lung pathological conditions and several routes of administration. The results suggest that the putative clinical application of TMX-302 in lung disorders should be examined with caution because of its direct pro-inflammatory effects. Moreover, in this context, TMX-306 seems to be comparatively more effective and safer, deserving further investigations in drug development particularly for silicosis.

## Author Contributions

TF, LM, RB, AA, AF, MB, and VC – acquisition and analysis of data, illustration, revision for intellectual content and final approval. MU – contributions to design of the work, acquisition and analysis of data, drafting of the work, and supervision and final approval; RM – contributions to design of the work, drafting of the manuscript, revising it critically for important intellectual content, supervision, and final approval. AB – revising it critically for important intellectual content, supervision, and final approval. PS – contributions to design of the work, illustration, critical revision, supervision, and final approval; MM – design of the work, illustration, drafting of the manuscript, supervision, and final approval.

## Conflict of Interest Statement

The author RM declares that he was employed by Telormedix as Head of Drug Development until the end of November 2015. The author AB declares that he was employed by Telormedix as Scientific Adviser until the end of March 2014. The other authors declare no conflict of interest.
