# Supplementary material for: Potential of PEGylated Toll-Like Receptor 7 Ligands for Controlling Inflammation and Functional Changes in Mouse Models of Asthma and Silicosis
Source: Front Immunol. 2016 Mar 11;7:95. doi: 10.3389/fimmu.2016.00095 (PMC4786742; doi:10.3389/fimmu.2016.00095)
Supplement: Supplementary file 1 [file Data_Sheet_1.DOCX]

Supplementary Material

Potential of PEGylated toll like receptor 7 ligands for controlling inflammation and functional changes in mouse models of obstructive and restrictive lung diseases

Tatiana Paula Ferreira Teixeira^1^, Lívia Lacerda Mariano^1^, Roberta Ghilosso Bortolini^1^, Ana Carolina Santos de Arantes^1^, Andrey Junior Fernandes^1^, Michelle Berni^2^, Valentina Cecchinato^2^, Mariagrazia Uguccioni^2^, Roberto Maj^3^, Patricia Machado Rodrigues e Silva^1^ and Marco Aurélio Martins^1*^

^1^Laboratory of Inflammation, Oswaldo Cruz Institute, FIOCRUZ, Rio de Janeiro, Brazil;

^2^Institute for Research in Biomedicine, Universitá della Svizzera Italiana, Bellinzona, Switzerland;

^3^Telormedix SA, Bioggio, Switzerland.

*** Correspondence:** Dr. Marco Aurélio Martins, Laboratory of Inflammation, Oswaldo Cruz Institute - FIOCRUZ, Av. Brasil 4365, Manguinhos - Rio de Janeiro 21045-900 – RJ - Brazil.

mmartins@ioc.fiocruz.br

# Supplementary Data

**Animals.** C57BL/6 mice were purchased from Harlan (Italy). C57BL/6 mice were maintained in the animal facility of the Institute for Research in Biomedicine and all procedures were approved by the veterinarian authorities from the local committee (Comitato etico cantonale del Ticino, Switzerland) with the authorization number TI17/2010. Age- and sex-matched mice (6-8 weeks) were randomly assigned to 2 groups which were injected intraperitoneally with physiological solution (*n*=5) or 200 nmoles of TMX-306 (*n*=5). After 24 hours, mice were sacrificed and cellular suspension was obtained by blood, spleen and bone marrow and analyzed by flow cytometry.

**Flow cytometric analysis.** For surface staining of mouse specimens, cell suspensions were incubated with Fc blocking antibody (Bioxcell, 2.4G2) to avoid unspecific Fc Receptor binding. After washing, the cells were incubated with the appropriate combination of the following antibodies: CD11b-PECy7 (M1/70, BioLegend®), Ly6G-PE (1A8, BD Biosciences), Ly6C-Biotin (AL-21, BD Biosciences), CD3-APC(17A2, BioLegend®), CD45R-B220-PerCP-Cy5.5 (RA3-6B2, eBiosciences). To detect anti-Ly6C-Biotin antibody binding, cells were subsequently stained with streptavidin-FITC (Dako). The samples were acquired with BD LSRFortessa (BD Biosciences), and the results were analyzed with FlowJo software (Tree Star, Inc.).

# Supplementary Figures and Tables

Initially, in order to assess if the PEGylated compound TMX-306 would impact *per se* on cell mobilization from the bone marrow as well as subsequent distribution in blood circulation and spleen, TMX-306 was injected intraperitoneally in mice. The results obtained indicate that this PEGylated analogue, at the dose of 200 nmoles/mouse, does not affect cell mobilization and compartmentalization in wild type mice **(Supplementary Figure 1)**.

## Supplementary Figures

**Supplementary Figure 1.** TMX-306 does not induce mobilization of immune cells in wild-type mice. Frequency of B cells, T cells, Monocytes, Macrophages and Neutrophils in the blood, spleen and bone marrow of mice injected intraperitoneally with physiological solution (Controls, n=5) or 200 nmoles of TMX-306 (n=5). Data are presented as mean values.
